# Metabolic Changes Induced by Cerebral Ischemia, the Effect of Ischemic Preconditioning, and Hyperhomocysteinemia

**DOI:** 10.3390/biom12040554

**Published:** 2022-04-08

**Authors:** Eva Baranovicova, Petra Hnilicova, Dagmar Kalenska, Peter Kaplan, Maria Kovalska, Zuzana Tatarkova, Anna Tomascova, Jan Lehotsky

**Affiliations:** 1 Biomedical Center BioMed, Jessenius Faculty of Medicine, Comenius University in Bratislava, Mala Hora 4, 036 01 Martin, Slovakia; eva.baranovicova@uniba.sk (E.B.); petra.hnilicova@uniba.sk (P.H.); anna.tomascova@uniba.sk (A.T.); 2 Department of Anatomy, Jessenius Faculty of Medicine, Comenius University in Bratislava, Mala Hora 4, 036 01 Martin, Slovakia; dagmar.kalenska@uniba.sk; 3 Department of Medical Biochemistry, Jessenius Faculty of Medicine, Comenius University in Bratislava, Mala Hora 4, 036 01 Martin, Slovakia; peter.kaplan@uniba.sk (P.K.); zuzana.tatarkova@uniba.sk (Z.T.); 4 Department of Histology and Embryology, Jessenius Faculty of Medicine, Comenius University in Bratislava, Mala Hora 4, 036 01 Martin, Slovakia; maria.kovalska@uniba.sk

**Keywords:** NMR spectroscopy, cerebral ischemia, metabolites, hyperhomocysteinemia, blood plasma, brain tissues

## Abstract

^1^H Nuclear Magnetic Resonance (NMR) metabolomics is one of the fundamental tools in the fast-developing metabolomics field. It identifies and quantifies the most abundant metabolites, alterations of which can describe energy metabolism, activated immune response, protein synthesis and catabolism, neurotransmission, and many other factors. This paper summarizes our results of the ^1^H NMR metabolomics approach to characterize the distribution of relevant metabolites and their alterations induced by cerebral ischemic injury or its combination with hyperhomocysteinemia in the affected tissue and blood plasma in rodents. A decrease in the neurotransmitter pool in the brain tissue likely follows the disordered feasibility of post-ischemic neurotransmission. This decline is balanced by the increased tissue glutamine level with the detected impact on neuronal health. The ischemic injury was also manifested in the metabolomic alterations in blood plasma with the decreased levels of glycolytic intermediates, as well as a post-ischemically induced ketosis-like state with increased plasma ketone bodies. As the 3-hydroxybutyrate can act as a likely neuroprotectant, its post-ischemic increase can suggest its supporting role in balancing ischemic metabolic dysregulation. Furthermore, the ^1^H NMR approach revealed post-ischemically increased 3-hydroxybutyrate in the remote organs, such as the liver and heart, as well as decreased myocardial glutamate. Ischemic preconditioning, as a proposed protective strategy, was manifested in a lower extent of metabolomic changes and/or their faster recovery in a longitudinal study. The paper also summarizes the pre- and post-ischemic metabolomic changes in the rat hyperhomocysteinemic models. Animals are challenged with hyperglycemia and ketosis-like state. A decrease in several amino acids in plasma follows the onset and progression of hippocampal neuropathology when combined with ischemic injury. The ^1^H NMR metabolomics approach also offers a high potential for metabolites in discriminatory analysis in the search for potential biomarkers of ischemic injury. Based on our results and the literature data, this paper presents valuable findings applicable in clinical studies and suggests the precaution of a high protein diet, especially foods which are high in Met content and low in B vitamins, in the possible risk of human cerebrovascular neuropathology.

## 1. Introduction

### 1.1. General Description of Ischemic/Reperfusion (IR) Animal Models

Cerebral ischemia is one of the major causes of death and long-term disability in most industrialized populations [1]. Animal models play an inevitable role in the research of the etiology of ischemic damage and protection. Experimental approaches to model human cerebral ischemia include reduction in global cerebral blood flow or focal occlusion of the middle cerebral artery, considered to be closest to human ischemic stroke. This paper summarizes the results of our experiments using the model of global ischemic damage developed by Pulsinelli by four-vessel occlusion (4VO), which provides reversible forebrain ischemia in rats with high reproducibility [2]. Its simplified version using two-vessel occlusion coupled with hypotension is appropriate for the study of chronic cerebral hypoperfusion [3].

### 1.2. Metabolomic Approach

In recent decades, metabolomics studies are gaining extensive popularity. The enormous development in technology and bioinformatics makes the research design and data analysis more accessible. Progress in other biochemical research fields has uncovered that metabolites act not only as intermediates and nutrients, but they are also very important mediators, signaling and regulatory molecules, neurotransmitters, osmolytes, modulators of immunity response, and influencers of epigenetic changes. In addition, single metabolites or their clusters are of high potential in discriminatory analysis in the search for potential disease biomarkers.

Metabolomic research is nowadays carried out on two basal instrumental platforms, mass spectrometry (MS) and NMR spectroscopy, where both of them have their pros and cons. NMR spectroscopy is characterized by very high reproducibility [4] but lower sensitivity in comparison to MS, which shows high sensitivity balanced with average reproducibility. NMR requires minimal sample preparation, as the entire sample can be analyzed in one measurement. Its fundamental features make NMR not optimal for targeted analysis. The situation differs in MS, where more complex sample preparation is required, and different additional chromatography techniques dependent on metabolites analyses have to be used for sample fractionation which results in higher costs per sample. However, MS is more appropriate for targeted metabolomics. Taking this into consideration, although NMR is not able to cover that broad spectrum of metabolites as MS does, its potential for evaluating the most abundant metabolites in the sample with high reproducibility makes this method predestinated for fundamental metabolomics studies. In biomedical research, the NMR metabolomics approach includes analyses of tissues in vivo (MRS) [5], ex vivo (magic angle spinning NMR) [6], and ^1^H NMR spectroscopy of biofluids or tissue extracts. This review aims to summarize the main findings in metabolomic alterations in the ischemic brain model as well as the hyperhomocysteinemic model in rodents via in vitro ^1^H NMR spectroscopy.

## 2. Metabolomic Alterations

### 2.1. Brain Tissues after Cerebral Ischemia Studied by the ^1^H NMR 

Seconds to minutes after a focal or global ischemic attack, metabolic rates are reduced and an energy crisis takes place. A complex of interconnected biochemical events arises that is not always linear, but often circular, sequential, or causal. During a period of reperfusion, delivery of oxygen and nutrients to support cellular metabolism is partially or fully re-established and potentially damaging side products of cellular metabolism are to be eliminated. Basal metabolic alterations were described in the long-term continuum of biochemical research; however, the detailed metabolomic profile of the post-ischemic alterations remained for a long time scarcely studied, likely due to the non-effective experimental approach.

As shown in many extensive studies, the ischemically induced excessive glutamate release from neurons known as ‘glutamate neurotoxicity’ is one of the major causes of post-ischemic cell damage [7]. Glutamate was detected to be elevated rapidly after the onset of ischemia and declined following reperfusion [8]. In addition to other cerebral damaging mechanisms, excitotoxic glutamate inhibits mTOR signaling and leads to exaggerated neuronal insulin resistance [9], making neurons more vulnerable to metabolic stress. This condition can likely result in the switch for the demand for other energy substrates to preserve cells viability under disordered tissue metabolism. Extracellular glutamate is rapidly taken up and converted to glutamine in the glutamate–glutamine cycle by glutamine synthetase [10] localized in astrocytes, or other cells of the astroglial family [11]. The high abundance of both glutamate and glutamine in brain tissues makes them predetermined to be easily detected by NMR methods.

As documented by us, ^1^H NMR analysis of tissue homogenates in our experiments showed decreased glutamate content in the 24 h period after global cerebral ischemia in the rat cortex and hippocampus [12], and similarly in the hippocampus and cortex in both female and male mice, 1 h after two-vessel occlusion (2VO) operation [13]. Glutamate levels were found to decrease also in the left ischemic hemisphere in 1, 3, 9, and 24 h after middle carotid artery occlusion (MCAO) [14,15], as well as 7 days after transient and permanent MCAO in rats [16]. These findings are complementary to the study by Kovalenko et al. who observed postischemic depletion of synaptic vesicles, the mail storage of neurotransmitters [17].

On the other hand, in our study using global cerebral ischemia in rats [13] as well as in another study using the MCAO model [14], was observed an increased level of glutamine, the main product of eliminating excessive extracellular glutamate. Glutamine is considered a metabolically protective agent in the astroglial cells for the regulation and acceleration of post-ischemic immunological response in the process of proposed neuroinflammation [18], as it serves as the primary fuel for the protective immune cells, lymphocytes, neutrophils, macrophages, and also shows an important role in cytokine production [19]. It was shown that after MCAO in mice, resident microglia rapidly proliferate, playing a prominent role in phagocytosis [20,21]. In post-ischemic conditions, inflammatory cells infiltrate the brain and exhibit distinct temporal profiles for microglia, neutrophils, T-cells, astrocytes, and NK cells, as reviewed in detail by Yan et al. [21].

Together with glutamate, inhibitory neurotransmitter GABA (gamma-aminobutyrate) is excessively deregulated in the extracellular space after the ischemic event [22]. Likewise, the post-ischemic decrease in its tissue concentration was observed in our study using in vitro ^1^H NMR in the cortex and hippocampus in the 4VO model in rats [12]. A similar observation was documented in permanent MCAO and transient MCAO in the ischemic hemisphere in rats [16], and in the contralateral hemisphere after MCAO in rats [15]. This is in line with previous studies documenting reduced GABA transmission in cerebral I/R damage, supporting the fact that disturbances in the GABA system take place in the complex pathology of cerebral I/R injury [23].

Many ^1^H NMR studies, including our own on global ischemic injury [12,13,14], consistently showed decreased levels of aspartate in the brain tissues in different ischemic models on rodents. Aspartate was considered for a long time to be (together with glutamate) a major excitatory transmitter in the brain; however, its role in neurotransmission was questioned a few years ago by Herring et al. [24]. The leaking of aspartate, as well as glutamate and GABA, into extracellular space after the ischemic event occurs apparently due to the destabilization and deterioration of the plasma membrane and blood–brain barrier (BBB) [25,26]. Thus, our ^1^H NMR metabolomics analysis can provide deeper and on-time data on the post-ischemic neurotransmitter alterations in the brain. This approach can achieve more precise knowledge of the functionality/efficiency of neurotransmission in the affected brain region.

Another functional aspect linked with the monitoring of a series of metabolite’s content, such as the level of N-acetyl aspartate (NAA) enables the indirect estimation of neuronal damage by the detection of its level. NAA is abundantly present and almost exclusively localized in neurons, and it is considered to be a marker of neuronal viability/health. Studies on long-term focal ischemia, transient ischemia, and brain injury without neuronal death detected post-ischemic decreased NAA levels by in vivo MRS. Remarkably, the content was able to recover [27], which suggested the position of NAA as a marker of neuronal functionality rather than neuronal density [28]. Reduced NAA levels are also thought of as a valuable marker of brain injury after stroke or hypoxia with the power of the disorder outcome prediction [27]. This was supported by the findings that decreased NAA levels in brain tissue extracts via ^1^H NMR spectroscopy were found; however, these findings were in different proportions in our four-vessel occlusion model of global ischemia and also in all above-mentioned animal models of cerebral ischemia [12,13,14,15,16,29].

Choline is essential for the synthesis of cell membranes lipids and facilitates restorative arteriogenesis in the ischemia-damaged brain parenchyma [30]. Choline can be synthesized de novo by virtually all animals, but the main source remains the diet. Choline availability is metabolically a crucial point to achieve accelerated membrane repair for the survival/regeneration of the neuronal cells following ischemic injury, and oral administration of choline showed a partial neuroprotective effect after cerebral ischemia [31]. In our experiments, we used ^1^H NMR spectroscopy, which allows the estimation of choline level in the tissue and therewith the tissue potential for proposed repair of ischemic damage. As detected in our study in the 4VO model in rats, it was found to be decreased in both brain areas—the cortex and hippocampus—for 24 h [12]; however, this was not proved in the focal MCAO models, likely due to the different animal models of cerebral ischemia [14,15].

Our experimental approach using the 4VO Pulsinelli model of ischemia enables us to recapitulate the results of NMR analysis of some metabolites in detail, but we identified and quantified also additional groups of metabolites—such as branched-chain amino acids (BCAAs), phenylalanine, tyrosine, fumarate, inosine, ascorbate, succinate, myo-inositol, taurine, and alanine—that may be targeted in brain tissue homogenates by ^1^H NMR spectroscopy [12]. All of these metabolites are participating in a broad spectrum of biochemical pathways—such as energy metabolism, compartmentalization of glutamate, nitrogen homeostasis, synthesis of neurotransmitters [32]—as well as serving as radical scavengers in the rat brain [33].

The most important observations based on our study and other ^1^H NMR studies on postischemic rodent brains are extensively summarized in Table 1. It is obvious that some variability exists in the post-ischemic metabolomic response in specific brain tissues, which is dependent upon used ischemia models, ischemia severity, time of reperfusion, type of animals, and some other factors. That considered, we would rather recommend the evaluation of concrete brain subregions than the whole hemisphere or even the whole brain, which may not be representative enough to obtain reliable results. This approach can also suppress brain region-specific differences that—although the brain regions show many common post-ischemic metabolomics features—were present and were shown and proved in our previous work between the cortex and hippocampus [12].

### 2.2. Circulating Metabolites in Animal Models of Cerebral Ischemia

The organism is a complex system functioning by the interaction of all organs, injury to one organ can impact the others and produce compensatory effects or secondary injury, which also applies to cerebral ischemia [37]. Blood serves as the main mediator and carrier in inter-organ communication and there is increasing evidence that brain ischemic injury induces not only cerebral regional metabolomic alterations, but also post-ischemic shifts in metabolite levels which were also observable in the blood [12,34,35,36,38]. ^1^H NMR spectroscopy as a very robust analytical methodology is, however, balanced by the reduced sensitivity. The methodology is suitable for analytical plasma metabolomics since it can detect about 70 of the most abundant metabolites [39], of which about 30 can be quantified responsibly and reliably, depending on the instrument, sample preparation, acquisition, etc.

The metabolomics alterations in blood plasma generally indicate a switch in the overall organism energy metabolism. Remarkably, MCAO in mice induced brain-remoted hepatic ketogenesis as evaluated via GC-MS [38]. Similarly, in our experiments, in the 4VO model of cerebral ischemia in rats, ^1^H NMR spectroscopy was able to detect a comparable increase in ketone bodies (3-hydroxybutyrate, acetoacetate, and acetone) in blood plasma in the 24 h reperfusion period [35]. This was accompanied by the increased plasma glucose level, in parallel with the decline of glycolytic intermediates pyruvate and lactate, indicating suppressed glycolysis [12,35]. In the other longitudinal study in this laboratory, we characterized the extent of metabolic changes in the 3 h, 24 h, and 72 h periods after the cerebral ischemic event induced by the 4VO model [34]. The study revealed that ketone bodies were the highest for 3 h after the ischemic event, continually decreasing but still not achieving the level of controls within the 72 h reperfusion period. On the other hand, blood glucose was steadily increasing with the time of reperfusion. Glycolytic intermediates, lactate, and pyruvate—after initial significant depletion—gradually leveled off towards the levels of controls. A very similar course was observed for alanine, which participates together with lactate and pyruvate in fundamental biochemical pathways: in Cahill’s and Cori´s cycles, the gluconeogenetic pathways. Branched-chain amino acids BCAAs (leucine, isoleucine, and valine) and their ketoacids-BCKAs, as important alternative energy substrates, showed a very similar peak-shaped course with a maximum in 24 h reperfusion period. Initially, decreased glutamine levels in blood plasma were restored on the third day after the ischemic event, to the levels of controls [34]. As generally accepted, cerebral ischemia activated the innate and adaptive immune system response and led to a massive migration of peripheral leukocytes in the brain, with a sequence of neutrophils first, followed by monocytes and lymphocytes [21]. All immunocompetent cells are glutamine dependent, which is essentially needed for their proliferation. Thus, accelerated post-ischemic immune response—combined with the compensatory role of renal used glutamine in the post-ischemic acidosis—may lead to the depletion of glutamine levels observed in our experiments in the ischemic/reperfused blood plasma.

Glutamate excitotoxicity that is induced by ischemic events resulted in many deleterious facets, including neuronal insulin desensitization [9], which limits the neurons in the utilization of glucose. Adaptative cerebral metabolism allows the use of alternative metabolic substrates that can fulfill metabolic requirements in periods of impaired glycolysis. Ketone bodies significantly contribute to cerebral metabolism and they can provide as much as 70% of the brain’s energy needs, energetically more efficiently than glucose [40]. As we showed, during the time of acute brain injury, cerebral uptake of ketones increased significantly [38,40], with additional elevation with the restored cerebral blood flow [41]. 3-hydroxybutyrate, besides serving as an energy substrate, showed an important neuroprotective effect, as it could protect neurons against glutamate-mediated apoptosis and necrosis [42], including the mechanism of the attenuation of the formation of reactive oxidant species [43]. In addition, 3-hydroxybutyrate was reported to help to enhance post-ischemically lowered GABA levels in brain tissues with the subsequent enhancement of GABA-mediated transmission [44,45]. To complete the complexity of the scheme, it was shown that the immediate oxidation of ketone bodies by the brain tissue induces a decrease in cerebral glucose uptake despite an adequate glucose supply to the brain [46,47]. As brain glucose utilization accounts for a significant part of whole-body glucose disposal, post-ischemically observed hyperglycemia may result from suppressed glucose utilization in the brain tissue, and/or the results of post-ischemic metabolic stress response activated by adrenal glands.

**Figure 1:**
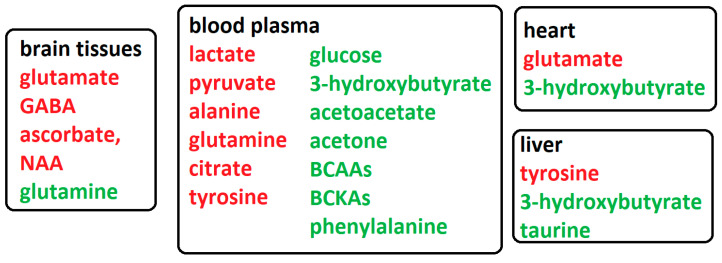
Schematic representation of the main metabolomic changes after cerebral ischemia in rodent models in brain tissues, blood plasma, and remote organs, as detected by ^1^H NMR (12,29,33,34,37); red—decreased levels; green—increased levels.

The detected switch in the energy metabolism towards accelerated production of ketone bodies by the extra-cerebrally localized liver seems to be programmed and important in the complex process to support recovery from ischemic brain damage. From a holistic point of view, it is obvious that cerebral ischemia induces changes not only in the affected tissue but also evokes systemic changes in the distinct organs. As shown on the second day after MCAO in rats, an increase in 3-hydroxybutyrate, taurine, and choline was detected in the liver, together with other changes [29]. Similarly, documented by us in this laboratory, cerebral ischemia induced an increase in the 3-hydroxybutyrate in homogenized heart tissue [12]. Furthermore, detected post-ischemic decrease in glutamate in the rat heart may further affect cardiac function, as the heart presents ionotropic [48] and metabotropic [49] glutamate receptors. The selected metabolomics alterations in organs and blood plasma after the 4VO model in cerebral ischemia are shown in Figure 1.

### 2.3. Possible ^1^H NMR Biomarkers of the Cerebral Ischemia

Recently, there have been two general approaches to analyzing metabolomic data. The first one, to explain biology, is based on significant differences between/among groups, where a hypothesis test resulting in a *p*-value is decisive. The second approach is to aim to identify biomarkers, molecules whose levels can be determined with optimal sensitivity/specificity, which requires another kind of statistical evaluation. Xia et al. recommended the use of the value of the area under the curve (AUC) derived from the ROC curve as a quantitative parameter to judge the discrimination performance [50]. Application of the metabolomics approach to the discovery of biomarkers in MCAO models in murine was reviewed very recently by Jia et al. [51]; however, the paper is concerned with upregulation or downregulation of metabolite levels, without assessment of the discriminatory power of metabolites. Very similarly, in the work by Wang et al., significantly changed metabolites after hypothesis testing without additional discriminatory analysis were claimed to serve as potential biomarkers in cerebrospinal fluid in rats after cerebral ischemia [52]. In our experiments, we employed the discriminatory algorithm random forest with the Monte-Carlo cross-validation using balanced subsampling (with two-thirds of samples to evaluate feature performance and one-third to validate the model), to uncover the ability to serve as a biomarker of cerebral ischemia in rodent models in both, the brain tissues homogenates and the blood plasma. In the hippocampus and cortex 24 h after cerebral ischemia by 4VO, we recognized metabolites that discriminated post-ischemic tissue from controls with parameter AUC = 1 [12]. From a practical point of view, much more interesting are biomarkers found in the blood plasma, which are due to their easy availability predestined for clinical use. As we explored, in the 4VO rodent model of cerebral ischemia, metabolomics changes were so strong that there were individual metabolites or their groups found that discriminated ideally with AUC = 1. All potential biomarkers are extensively summarized in Table 2. It is noteworthy that metabolites marked as biomarkers after ideal discrimination are rather to be considered non-specific biomarkers since they could overlap with other diseases/injuries. Based on data showing the extent of metabolomic alterations regarding ischemia severity/completeness in brain tissue, heart tissue, as well as in blood [12], the intensity of ischemia considerably influences the discriminatory power of particular metabolites which additionally indicates the high impact of disease severity to all organs.

### 2.4. ^1^H NMR Metabolomics Approach in the Ischemic Preconditioning (IPC)

By the adaptation of an organism to a sublethal ischemic injury, the organism can obtain some level of resistance against subsequent ischemic attack. The animals that underwent ischemic preconditioning generally showed a higher number of surviving neurons in brain structures after subsequent ischemia [53]. The protective effect of IPC is linked to various mechanisms, such as reduction in cellular apoptosis [54], a decrease in neurovascular damage [55], a decrease in the inflammatory response [56], and metabolomic reprogramming [57], however, the exact mechanism is still not completely understood.

In our metabolomics studies in the 4VO model of global cerebral ischemia, the effect of ischemic preconditioning was generally manifested in (i) the lower extent of post-ischemic metabolites alterations and (ii) faster metabolomics recovery towards the levels of controls in the ischemic tissue This was proved for the metabolites: glutamate, GABA, aspartate, myo-inositol detected in cortex and metabolites: glutamate, GABA, ascorbate, inosine, choline, and myo-inositol detected in the hippocampus in 24 h reperfusion after 4VO [12], however, the level of NAA, as a proposed indicator of neuronal viability did not fully reflect the introduction of the IPC maneuver [12].

In blood plasma, the effect of IPC was obvious for metabolites: ketone bodies, BCAAs, BCKAs, lactate, pyruvate, and phenylalanine [12,34,35]. Interestingly, blood glucose level was post-ischemically elevated in 3 h, 24 h, and 72 h in animals after 4VO, but not in IPC animals in any reperfusion period, which may have a significant effect not only on the energy metabolism of the affected organism but also on the general tissue and organism post-ischemic recovery, since hyperglycemia is a known unfavorable condition in ischemic output also in the human pathology [58].

### 2.5. ^1^H NMR Approach in Animal Models of Hyperhomocysteinemia and Cerebral Ischemia with Induced Hyperhomocysteinemia

Many experimental and clinical studies show that co-morbid disorders are risk factors for developing human vascular pathologies, such as stroke and cardiac failure [59,60]. Mild hyperhomocysteinemia (hHcy) may increase the risk of these pathologies, probably due to the pleiotropic biochemical properties of homocysteine (Hcy) [59,61,62]. Hcy is a critical component of the one-carbon methionine (Met) metabolism [63,64]. Its toxicity is the result of auto-oxidation and free radical generation [61,64] as well as the toxicity of its metabolic products, such as Hcy-thiolactone and homocysteic acid. Hyperhomocysteinemia in humans can be caused by a high intake of methionine, deficiency of vitamin B12 and folate, or genetic polymorphism of metabolic genes. Hyperhomocysteinemia-induced oxidative stress, inflammation, endoplasmic reticulum stress, and likely disorders of mitochondria all play an important role in the pathogenesis of acute and degenerative neurological diseases. Remarkably, pyramidal neurons of the hippocampus are sensitive to prolonged levels of homocysteine due to the absence of metabolization by transsulfuration, as well as by folate or B12-dependent remethylation. The role of hyperhomocysteinemia in amyloid deposition and hyperphosphorylation of tau protein in the brain, along with plasma metabolic alterations in cerebral ischemia–reperfusion injury, was noticed by our experiments and other studies [59,60]. Prevention of hyperhomocysteinemia may have therapeutic implications in cerebral ischemic stroke and deserves investigation as related pathomechanisms are still not well characterized and are thought to be complex and multifactorial [65]. It is believed that, in parallel to the induction of cellular and molecular injury induced by Hcy, an impairment of epigenetic control mechanisms of gene expression due to the interference with one-carbon unit metabolism or the changes in the structure and function of proteins by the post-translational N- and S-homocysteinylation can explain Hcy toxicity.

Transport of Hcy across the plasma membrane is mediated by several amino acid transport systems from the solute carrier (SLC) superfamily, including sodium-dependent systems for aspartate and glutamate (XAG), alanine–serine–cysteine (ASC), and alanine (A), and sodium-independent large neutral branched-chain or aromatic amino acid system L [66,67]. This is the reason why an elevated level of Hcy can concur or limit the transport of many amino acids into the tissues, including the brain. This can heavily impact relevant intra-tissue levels of respective amino acids or amino acids blood plasma distribution, which can be detected by the ^1^H NMR approach.

Experimental models of hyperhomocysteinemia (hHcy) widely utilize per os or intraperitoneal Hcy administration [34]. The application of the high (supraphysiological) methionine diet is an alternative approach, which leads to hHcy conditions. A particular link between the hHcy and the glucose metabolism in animal models can be expected, since hHcy mice showed higher insulin levels [68]; in another way, the physiological hyperinsulinemia increases Met-Hcy clearance [69]. In our experimental settings, the ^1^H NMR metabolomics analysis revealed that rats on high methionine diet (hyperhomocysteinemic conditions) were challenged with hyperglycemia and a ketotic-like state [70]. The relative levels of plasma amino acids: phenylalanine, tryptophan, tyrosine, and histidine were significantly decreased in Met overfed rats [71], and relative plasma levels of BCAAs were decreased also in hHcy induced by Hcy intraperitoneal application in a rat model [36]. Remarkably, altered plasma metabolome in the hHcy conditions affects also the onset and possible progression of hippocampal neuropathology, as well as the behavioral pattern in rats.

Unfortunately, only spare information is provided on the effect of hHcy at the level of metabolic changes in vivo [66,72,73,74] and we used a novel approach such as in vivo magnetic resonance spectroscopy (MRS) for the measurement of several metabolites, such as total N-acetyl aspartate (tNAA), myo-inositol (mIns), total choline (tCho), and total creatine (tCr) containing compounds in the hippocampus of the rats on high methionine diet (hHcy condition) [73]. When expressed as ratios, we found decreased tNAA (an index of axonal integrity)/tCr, tNAA/mIns, and also reduced mlns/tCr levels. An increase in tCho/tNAA and tCho/tCr levels was also detected. Although the changes did not express high statistical significance, an obvious increase (more than 10%) in the hippocampal volume was detected in animals with high methionine diet over the threshold of the normal tissue volume without methionine pretreatment. This cytotoxic hippocampal edema can be ascribed to the partial disruption of the blood–brain barrier caused by the incremental Hcy or its metabolites, as well as by the direct Hcy excitotoxic effect on astrocytes. [64]. Impairment of the endothelium’s capacity to regulate vascular tone by reduced bioavailability of NO and endothelial dysfunction follows hHCy conditions [75,76] and in turn affects adjacent astrocytes to modulate extracellular space volume.

Regarding the biological significance of individual metabolites which was measured in the hHcy conditions, it is noted that tCr has the most stable concentration among ^1^H MRS-detectable metabolites in the brain, and thus it is used as a reference for relative metabolite quantification [77]. Cr as a marker of glial cells and its simultaneous occurrence with the neuroaxonal marker tNAA and other glial components, tCho (mainly oligodendrocytes) and mIns (mainly astrocytes), enables us to non-invasively reflect the intracranial metabolic changes [78]. Hyperhomocysteinemic metabolic injury resulting in the proposed axonal dysfunction is detectable by the reduced tNAA (one of the most common amino acids in the brain which acts as an important organic osmolyte, and a precursor for myelin-lipids synthesis) and reflects pathologic severity [79], which correlate with clinical measures in cross-sectional studies [80]. Choline, as a precursor of membrane metabolism, is considered a ^1^H MRS marker of membrane density, myelin sheet degradation, ongoing gliosis, and ischemic and re/de-myelinization processes [81,82]. The rise in its ratio, as was detected in our experiments, can finalize to the above-mentioned processes also in hHcy conditions. MIns, as an important osmolyte, is synthesized in glial cells, representing glia proliferation. Its decreased ratios revealed in our experiments are in the line of previous studies indicating neuronal tissue breakdown, edema, and/or cell lysis, or a shift from oxidative energy metabolism due to the mitochondrial dysfunction induced by hHcy. Met-Hcy cycle is connected to one-carbon metabolism and editing processes interfering with epigenetic control and proteosynthesis [83]. In addition, experiments proved that a high methionine diet (hHCy conditions) produces a toxic environment, changed metabolic ratio, and hippocampal volume, manifested also in our study by the altered histopathological and behavioral pattern in rats [74]. All these results are in the line with the valuable findings applicable in clinical studies and suggest the precaution of a high protein diet (especially food with high Met content and low B vitamins) in the possible risk of human cerebrovascular neuropathology.

Mitochondria as an essential cellular energy producer, biosynthetic, and catabolic organelle regulates cell survival and death [84]. Its function is critically required for the energetic and metabolic processes in the cerebrovascular and cardiovascular systems, and mitochondrial disorders play an important role in the etiology of cerebral and cardiovascular diseases [85]. Remarkably, hyperhomocysteinemic conditions deeply affect rat heart function to the depression of left ventricular developed pressure, maximal rates of contraction (+dP/dt) and relaxation (−dP/dt), depressed activities of electron transport chain (ETC) complexes II–IV [85]. In our study using induction of hHcy by intraperitoneal injection of homocysteine, we did not show elevation of protein oxidative damage, as detected by unchanged protein carbonyl, thiol, and dithyrosine contents and depressed level of protein adducts with 4-hydroxynonenal. However, mass spectrometry revealed eight proteins with elevated expression playing roles in the cellular stress response, bioenergetics, and redox balance. Results of our experiments suggest that respiratory dysfunctions are not causally linked with oxidative damage of proteins [86], but functional alterations manifested by cardiac dysfunction were linked with the significant reduction in sarcoplasmic reticular Ca^2+^-handling proteins such as Ca^2+^-ATPase (SERCA2), calsequestrin, and histidine-rich calcium-binding protein with the unchanged level of regulatory protein phospholamban (PLN). The increased PLN:SERCA2 ratio observed in our study resulted in the inhibition of the Ca^2+^ ATPase activity at low free Ca^2+^ concentrations [86]. Notably, hHcy is manifested in cardiac tissue by the impaired heart contractility as a result of mitochondrial energy deterioration and disturbances in sarcoplasmic Ca^2+^ handling [87]. Much less is known regarding the effect of hHcy on the tricarboxylic acid cycle (TCA), the final common pathway for the oxidation of major nutrients. Hcy or its metabolites in rats did not change the activity of citrate synthase; however, it inhibits activity and decreases the level of the second enzyme, aconitase, which is highly susceptible to ROS-induced damage. Notably, succinate dehydrogenase (SDH)—which unifies the metabolism of branched-chain amino acids and TCA—was found to be upregulated, indicating a possible adaptive response to the mild hHcy, at least in the rat heart [86]. Conclusively, we showed that hHcy induced heart dysfunction leads to the alterations in the energy-linked metabolites in the cardiac mitochondria and tissue parenchyma, which is eventually reflected in the distributed pattern of metabolites in the blood plasma. Thus, the level of individual metabolites in blood plasma reflect the complex response of the organism to the hyperhomocysteinemic conditions and additionally can also be influenced by the ischemic injury in the remote brain organ (Table 1).

In a series of experiments, we compared the pattern of blood plasma metabolites detected by ^1^H NMR in rats with induced global cerebral ischemia with the rats with induced hyperhomocysteinemia combined with successive global ischemia. The analysis showed that both conditions resulted in very similar patterns in the alterations of plasma metabolites as was observed in not-Hcy-treated animals (see Table 2). An increase in 3-hydroxybutyrate, acetone, phenylalanine, and BCAAs was compensated by a decrease in pyruvate, citrate, and triacylglycerols [36] as a large part of the lipoprotein fraction [88]. The main difference observed between the hHcy and the non-hHcy ischemic animals was the post-ischemic decrease in glucose plasma level [36], which indicates the pronounced deregulation of glucose metabolism in the hyperhomocysteinemic condition. However, the additional impact of ischemic attack aggravates hHcy-induced neurodegenerative processes observed by the histological patterns and eventually leads to the manifestation of the development of Alzheimer’s disease-like neuropathology [89]. The delayed post-ischemic metabolic recovery of hHcy animals may, furthermore, restrict protein and neurotransmitters synthesis, nitrogen homeostasis, and other healing processes since hHcy alone (without induction of ischemia) restricts the amount of many essential and non-essential amino acids in the rat blood plasma, which is believed as a main metabolomic supply for the brain tissue. Since many amino acids use common BBB transporters [90] to enter the brain parenchyma, their imbalanced levels and relations in the blood plasma may result in further competition when crossing BBB and thus more influence the metabolomic environment in the brain during ischemic recovery in hHcy animals.

## 3. Challenges to Clinical Translation and Future Perspectives

The advancement of basic medical research to materialize the ‘bench-to-bedside’ concept is inevitably linked with the therapy progress of cerebrovascular diseases. Proper analysis of metabolites content with their proven many biological functions—such as modifying proteins and genes, serving as signal molecules, neurotransmitters, osmolytes, supplying cell energy, and so on—should not be underestimated. The excellent feature of metabolomic studies to reveal a fast response to ischemic insult makes it preferential in comparison to proteomic or histologic findings on the affected tissue that can evolve over several days. Based on all studies reviewed, including ours, it seems that cerebral ischemia occurs on the level of the whole organism, with its own adapting/repairing mechanisms observable at the metabolomic level. The most obvious is the conversion of excessive glutamate to glutamine to accelerate organism immune response induction of synthesis of neuroprotective liver ketones, which are able to pass the blood–brain barrier and target nerve tissue. It seems that early intervention towards metabolomics pathways can have deeper benefits for the injured tissue and also later manifest at other levels. Considering the fact that basal metabolomic pathways are relatively well described and understood from years of biochemical research, studies describing influence towards suppressing/enhancing metabolism are achievable.

Limitations of experimental studies often include animal models of cerebral ischemia which are conducted on young animals without any comorbidity. This results in a net injury effect, but the ischemic damage in the context of often chronic diseases found in the elderly human population is, in this way, not documented. The extension of experimental models by other risk factors prevalent in civilization makes information obtained better translatable to humans. The model of hyperhomocysteinemia as a recognized cerebrovascular risk factor widens our understanding of the proposed coincidence with ischemic injury, and it should be investigated in further detail in parallel with obesity, diabetes, and other conditions. The future of the ^1^H NMR technique in the metabolomic studies of cerebral ischemia depends on the possibility of broadening the spectrum of validated animal models, including not only risk factors and unfavorable conditions, but also the protective agents and schemas. Besides that, it should stand a chance to support the results by complementary biochemical methods. In any case, the ^1^H NMR metabolomic studies should not only be purely descriptive, but the authors should try the interpretation of results and assessment of observation in a holistic context of ischemic injury affecting the whole organism, and then the metabolomic studies will gain their values.

## 4. Conclusions

^1^H NMR metabolomics is a suitable method to track changes in the levels of most abundant metabolites present in the ischemically affected tissues and blood plasma. Studies document that observed changes were more or less consistent among various models of cerebral ischemia: in the brain tissue, the decreased levels of glutamate, GABA, NAA, choline, and aspartate balanced with the increased glutamine level. Remarkably, cerebral ischemia is manifested also in the metabolomics changes in remote organs, such as the heart and liver, and also in the blood plasma, where probably the most pronounced change was post-ischemically induced hyperglycemia with the ketosis-like state. The changes that were not discussed in detail are summarized and referred to. Ischemically preconditioned animals generally showed a lower extent of metabolomic changes or faster recovery in the longitudinal studies. The paper also discusses the metabolomic changes in the hyperhomocysteinemia models in rats. A combination of hHcy with cerebral ischemia induced more pronounced alterations in the metabolomic state manifested by the apparent decline in the levels of many amino acids. Concurrent hyperglycemia may further handicap animals in the post-ischemic recovery and additionally result in deeper and exaggerated histological and functional neurodegenerative features. Finally, data obtained by the ^1^H NMR spectroscopy approach on animal models of cerebral ischemia showed high potential in the search for biomarkers of ischemic injury and these must be thoroughly elaborated for experimental and clinical purposes.

## Figures and Tables

**Table 1:** Metabolomic changes in tissues and blood plasma in different animal models of cerebral ischemia by in vitro ^1^H NMR metabolomic approach

		Reper-Fusion		Increase	Decrease	References
4VO	rats	24 h	cortex	glutamine, isoleucine, valine, phenylalanine, fumarate	glutamate, GABA, NAA, choline	[12]
		24 h	hippocampus		glutamate, GABA, ascorbate, inosine, NAA, choline, myo-inositol	[12]
		3 h	blood plasma	glucose, 3-hydroxybutyrate, acetoacetate, BCAAs, BCKAs, phenylalanine	lactate, pyruvate, alanine, citrate, glutamine, lipoproteins, tyrosine, lysine	[34]
		24 h	blood plasma	glucose, 3-hydroxybutyrate, acetoacetate, acetone, BCAAs, BCKAs, phenylalanine	lactate, pyruvate, alanine, citrate, glutamine, lipoproteins, citrate	[34,35]
		72 h	blood plasma	glucose	citrate	[34]
		24 h	heart	3-hydroxybutyrate	glutamate	[12]
2VO	mice female	1 h	cortex	leucine, isoleucine, valine, alanine, lysine, glutamine, succinate, myo-inositol, GABA	glutamate, aspartate, taurine, NAA	[13]
		1 h	hippocampus	leucine, isoleucine, valine, alanine, GABA, tyrosine	glutamate, aspartate	[13]
	mice male	1 h	cortex	leucine, isoleucine, valine, alanine, lysine, GABA, glutamine, tyrosine	glutamate, aspartate, NAA,	[13]
		1 h	hippocampus	isoleucine, valine. alanine, GABA, glutamine	glutamate	[13]
MCAO	rat	24 h	left ischemic cerebral hemisphere	glycine, GABA, alanine, choline	myo-inositol, NAA, aspartate, glutamate, creatine	[14]
		24 h	right cerebellum	GABA, aspartate, glutamine, choline	glutamate, succinate, creatine	[14]
MCAO	rat	1 h	ipsilateral (ischemic) hemisphere	alanine, GABA, choline, glycine	NAA, glutamate, creatine	[15]
		3 h	ipsilateral (ischemic) hemisphere	alanine, GABA, choline, glycine	NAA, glutamate, aspartate, creatine	[15]
		9 h	ipsilateral (ischemic) hemisphere	alanine, glycine	NAA, glutamate, aspartate, creatine	[15]
		24 h	ipsilateral (ischemic) hemisphere	alanine, GABA, choline, glycine	NAA, glutamate, aspartate, creatine	[15]
	rat	1 h	contralateral hemisphere	alanine, aspartate	GABA, glutamate	[15]
		3 h	contralateral hemisphere	aspartate, glycine		[15]
		9 h	contralateral hemisphere	aspartate		[15]
		24 h	contralateral hemisphere	alanine, aspartate, choline	creatine	[15]
pMCAO	rat	7 days	left brain extracts	glutamine	NAA, GABA, glutamate, succinate	[16]
tMCAO	rat	2 h–7 days	left brain extracts		NAA, GABA, glutamate, succinate	[16]
MCAO	rat 3 months	day 2	brain	lysine, tryptophan, glycine, fumarate	NAA	[29]
	rat 12 months	day 2	brain		NAA	[29]
	rat 3 months	day 2	liver			[29]
	rat 12 months	day 2	liver	choline, 3-hydroxybutyrate, taurine	tyrosine, tryptophan, serine, alanine, histamine	[29]
	rat 3 months	day 2	blood plasma	lysine	tyrosine, choline, citrate	[29]
	rat 12 months	day 2	blood plasma	lysine, isoleucine	tyrosine, citrate, proline, threonine	[29]
4VO	rat-hHcy	24 h	blood plasma	lactate, leucine, isoleucine, valine, acetone, 3-hydroxybutyrate, phenylalanine, creatine	glucose, pyruvate, citrate, lipoproteins	[36]

**Table 2:** Discrimination analysis towards biomarkers in the 4VO animal model of cerebral ischemia in rats, metabolite levels evaluated by ^1^H NMR spectrometry

Reperfusion	Medium	Metabolites Discriminating Ischemic Rats against Controls with AUC Value Obtained after Discrimination	References
24 h	cortex	fumarate 1, choline 0.998, phenylalanine 0.997, valine 0.995, isoleucine 0.925, glutamine 0.923, alanine 0.842, GABA 0.809	[12]
24 h	hippocampus	glutamate 1, GABA 1, choline 1, myo-inositol 0.978, ascorbate 0.963, inosine 0.919, NAA 0.918, creatine 0.894, succinate 0.878	[12]
3 h	blood plasma	alanine, phenylalanine, acetoacetate, all in combination AUC = 1	[34]
24 h	blood plasma	3-hydroxybutyrate, acetoacetate, leucine, pyruvate, choline, all in combination AUC = 1	[35]
24 h	blood plasma	alanine, isoleucine, leucine acetoacetate, pyruvate, all in combination AUC = 1	[34]
72 h	blood plasma	acetoacetate, lysine, 3-hydroxybutyrate, glucose, citrate, all in combination AUC = 0.96	[34]
24 h	heart	glutamate 0.898	[12]
24 h	blood plasma, hHcy rats	citrate, 3-hydroxybutyrate, creatine, glucose, all in combination AUC = 1	[36]

## Data Availability

The data that originated from our group are available on request: eva.baranovicova@uniba.sk.

## References

[1] Bonita R. (1992). Epidemiology of Stroke. Lancet.

[2] Pulsinelli W.A., Brierley J.B. (1979). A New Model of Bilateral Hemispheric Ischemia in the Unanesthetized Rat. Stroke.

[3] Traystman R.J. (2003). Animal Models of Focal and Global Cerebral Ischemia. ILAR J..

[4] Wishart D.S. (2019). NMR Metabolomics: A Look Ahead. J. Magn. Reson..

[5] Tognarelli J.M., Dawood M., Shariff M.I.F., Grover V.P.B., Crossey M.M.E., Cox I.J., Taylor-Robinson S.D., McPhail M.J.W. (2015). Magnetic Resonance Spectroscopy: Principles and Techniques: Lessons for Clinicians. J. Clin. Exp. Hepatol..

[6] Hu J.Z. (2016). Magic Angle Spinning NMR Metabolomics. Metab. Open Access.

[7] Choi D.W., Rothman S.M. (1990). The Role of Glutamate Neurotoxicity in Hypoxic-Ischemic Neuronal Death. Annu. Rev. Neurosci..

[8] Nishizawa Y. (2001). Glutamate Release and Neuronal Damage in Ischemia. Life Sci..

[9] Pomytkin I., Krasil’nikova I., Bakaeva Z., Surin A., Pinelis V. (2019). Excitotoxic Glutamate Causes Neuronal Insulin Resistance by Inhibiting Insulin Receptor/Akt/MTOR Pathway. Mol. Brain.

[10] Rose C.F., Verkhratsky A., Parpura V. (2013). Astrocyte Glutamine Synthetase: Pivotal in Health and Disease. Biochem. Soc. Trans..

[11] Anlauf E., Derouiche A. (2013). Glutamine Synthetase as an Astrocytic Marker: Its Cell Type and Vesicle Localization. Front. Endocrinol..

[12] Baranovicova E., Kalenska D., Grendar M., Lehotsky J. (2021). Metabolomic Recovery as a Result of Ischemic Preconditioning Was More Pronounced in Hippocampus than in Cortex That Appeared More Sensitive to Metabolomic Blood Components. Metabolites.

[13] Zhang T., Wang W., Huang J., Liu X., Zhang H., Zhang N. (2016). Metabolomic Investigation of Regional Brain Tissue Dysfunctions Induced by Global Cerebral Ischemia. BMC Neurosci..

[14] Hu Z.-L., Xia H.-H., Yang Y.-J., Zheng H., Zhao L.-C., Chen Y.-C., Zhuge Q.-C., Xia N.-Z., Gao H.-C., Chen W.-J. (2018). Metabolic Alterations in the Rat Cerebellum Following Acute Middle Cerebral Artery Occlusion, as Determined by 1H NMR Spectroscopy. Mol. Med. Rep..

[15] Ruan L., Wang Y., Chen S., Zhao T., Huang Q., Hu Z., Xia N., Liu J., Chen W., Zhang Y. (2017). Metabolite Changes in the Ipsilateral and Contralateral Cerebral Hemispheres in Rats with Middle Cerebral Artery Occlusion. Neural Regen. Res..

[16] Huang Q., Li C., Xia N., Zhao L., Wang D., Yang Y., Gao H. (2018). Neurochemical Changes in Unilateral Cerebral Hemisphere during the Subacute Stage of Focal Cerebral Ischemia–reperfusion in Rats: An Ex Vivo 1H Magnetic Resonance Spectroscopy Study. Brain Res..

[17] Kovalenko T., Osadchenko I., Nikonenko A., Lushnikova I., Voronin K., Nikonenko I., Muller D., Skibo G. (2006). Ischemia-Induced Modifications in Hippocampal CA1 Stratum Radiatum Excitatory Synapses. Hippocampus.

[18] Colombo E., Farina C. (2016). Astrocytes: Key Regulators of Neuroinflammation. Trends Immunol..

[19] Newsholme P., Curi R., Pithon Curi T.C., Murphy C.J., Garcia C., Pires de Melo M. (1999). Glutamine Metabolism by Lymphocytes, Macrophages, and Neutrophils: Its Importance in Health and Disease. J. Nutr. Biochem..

[20] Denes A., Vidyasagar R., Feng J., Narvainen J., McColl B.W., Kauppinen R.A., Allan S.M. (2007). Proliferating Resident Microglia after Focal Cerebral Ischaemia in Mice. J. Cereb. Blood Flow Metab..

[21] Yan T., Chopp M., Chen J. (2015). Experimental Animal Models and Inflammatory Cellular Changes in Cerebral Ischemic and Hemorrhagic Stroke. Neurosci. Bull..

[22] Hutchinson P.J., O’Connell M.T., Al-Rawi P.G., Kett-White C.R., Gupta A.K., Maskell L.B., Pickard J.D., Kirkpatrick P.J. (2002). Increases in GABA Concentrations during Cerebral Ischaemia: A Microdialysis Study of Extracellular Amino Acids. J. Neurol. Neurosurg. Psychiatry.

[23] Chen C., Zhou X., He J., Xie Z., Xia S., Lu G. (2019). The Roles of GABA in Ischemia–reperfusion Injury in the Central Nervous System and Peripheral Organs. Oxid. Med. Cell. Longev..

[24] Herring B.E., Silm K., Edwards R.H., Nicoll R.A. (2015). Is Aspartate an Excitatory Neurotransmitter?. J. Neurosci..

[25] Phillis J.W., Smith-Barbour M., Perkins L.M., O’Regan M.H. (1994). Characterization of Glutamate, Aspartate, and GABA Release from Ischemic Rat Cerebral Cortex. Brain Res. Bull..

[26] Phillis J.W., O’Regan M.H. (1996). Mechanisms of Glutamate and Aspartate Release in the Ischemic Rat Cerebral Cortex. Brain Res..

[27] Demougeot C., Garnier P., Mossiat C., Bertrand N., Giroud M., Beley A., Marie C. (2001). N-Acetylaspartate, a Marker of Both Cellular Dysfunction and Neuronal Loss: Its Relevance to Studies of Acute Brain Injury. J. Neurochem..

[28] Schuff N., Meyerhoff D.J., Mueller S., Chao L., Sacrey D.T., Laxer K., Weiner M.W. (2006). *N*-Acetylaspartate as a Marker of Neuronal Injury in Neurodegenerative Disease. Adv. Exp. Med. Biol..

[29] Wesley U.V., Bhute V.J., Hatcher J.F., Palecek S.P., Dempsey R.J. (2019). Local and Systemic Metabolic Alterations in Brain, Plasma, and Liver of Rats in Response to Aging and Ischemic Stroke, as Detected by Nuclear Magnetic Resonance (NMR) Spectroscopy. Neurochem. Int..

[30] Jin X., Wang R., Wang H., Long C., Wang H. (2015). Brain Protection against Ischemic Stroke Using Choline as a New Molecular Bypass Treatment. Acta Pharmacol. Sin..

[31] Borges A.A., El-Batah P.N., Yamashita L.F., Santana A.d.S., Lopes A.C., Freymuller-Haapalainen E., Coimbra C.G., Sinigaglia-Coimbra R. (2015). Neuroprotective Effect of Oral Choline Administration after Global Brain Ischemia in Rats. Nutr. Neurosci..

[32] Fernstrom J.D. (2005). Branched-Chain Amino Acids and Brain Function. J. Nutr..

[33] Kasparová S., Brezová V., Valko M., Horecký J., Mlynárik V., Liptaj T., Vancová O., Ulicná O., Dobrota D. (2005). Study of the Oxidative Stress in a Rat Model of Chronic Brain Hypoperfusion. Neurochem. Int..

[34] Baranovicova E., Kalenska D., Tomascova A., Holubcikova S., Lehotsky J. (2020). Time-Related Metabolomics Study in the Rat Plasma after Global Cerebral Ischemia and Reperfusion: Effect of Ischemic Preconditioning. IUBMB Life.

[35] Baranovicova E., Grendar M., Kalenska D., Tomascova A., Cierny D., Lehotsky J. (2018). NMR Metabolomic Study of Blood Plasma in Ischemic and Ischemically Preconditioned Rats: An Increased Level of Ketone Bodies and Decreased Content of Glycolytic Products 24 h after Global Cerebral Ischemia. J. Physiol. Biochem..

[36] Baranovicova E., Kalenska D., Tomascova A., Lehotsky J. (2018). Metabolomic Study of Altered Energy Metabolism during Global Forebrain Ischemia and Ischemic Precoditioning in Blood Plasma in Homocysteine Treated Rats. J. Physiol. Pharmacol. Off. J. Pol. Physiol. Soc..

[37] Ma S., Zhao H., Ji X., Luo Y. (2015). Peripheral to Central: Organ Interactions in Stroke Pathophysiology. Exp. Neurol..

[38] Koch K., Berressem D., Konietzka J., Thinnes A., Eckert G.P., Klein J. (2017). Hepatic Ketogenesis Induced by Middle Cerebral Artery Occlusion in Mice. J. Am. Heart Assoc..

[39] Nagana Gowda G.A., Gowda Y.N., Raftery D. (2015). Expanding the Limits of Human Blood Metabolite Quantitation Using NMR Spectroscopy. Anal. Chem..

[40] White H., Venkatesh B. (2011). Clinical Review: Ketones and Brain Injury. Crit. Care Lond. Engl..

[41] Svart M., Gormsen L.C., Hansen J., Zeidler D., Gejl M., Vang K., Aanerud J., Moeller N. (2018). Regional Cerebral Effects of Ketone Body Infusion with 3-Hydroxybutyrate in Humans: Reduced Glucose Uptake, Unchanged Oxygen Consumption and Increased Blood Flow by Positron Emission Tomography. A Randomized, Controlled Trial. PLoS ONE.

[42] Prins M.L. (2008). Cerebral Metabolic Adaptation and Ketone Metabolism after Brain Injury. J. Cereb. Blood Flow Metab..

[43] Ziegler D.R., Ribeiro L.C., Hagenn M., Siqueira I.R., Araújo E., Torres I.L.S., Gottfried C., Netto C.A., Gonçalves C.-A. (2003). Ketogenic Diet Increases Glutathione Peroxidase Activity in Rat Hippocampus. Neurochem. Res..

[44] Gasior M., Rogawski M.A., Hartman A.L. (2006). Neuroprotective and Disease-Modifying Effects of the Ketogenic Diet. Behav. Pharmacol..

[45] Yudkoff M., Daikhin Y., Nissim I., Lazarow A., Nissim I. (2001). Ketogenic Diet, Amino Acid Metabolism, and Seizure Control. J. Neurosci. Res..

[46] LaManna J.C., Salem N., Puchowicz M., Erokwu B., Koppaka S., Flask C., Lee Z. (2009). KETONES SUPPRESS BRAIN GLUCOSE CONSUMPTION. Adv. Exp. Med. Biol..

[47] Hasselbalch S.G., Madsen P.L., Hageman L.P., Olsen K.S., Justesen N., Holm S., Paulson O.B. (1996). Changes in Cerebral Blood Flow and Carbohydrate Metabolism during Acute Hyperketonemia. Am. J. Physiol..

[48] Gill S.S., Pulido O.M., Mueller R.W., McGuire P.F. (1998). Molecular and Immunochemical Characterization of the Ionotropic Glutamate Receptors in the Rat Heart. Brain Res. Bull..

[49] Gill S.S., Pulido O.M., Mueller R.W., McGuire P.F. (1999). Immunochemical Localization of the Metabotropic Glutamate Receptors in the Rat Heart. Brain Res. Bull..

[50] Xia J., Broadhurst D.I., Wilson M., Wishart D.S. (2013). Translational Biomarker Discovery in Clinical Metabolomics: An Introductory Tutorial. Metabolomics Off. J. Metab. Soc..

[51] Jia J., Zhang H., Liang X., Dai Y., Liu L., Tan K., Ma R., Luo J., Ding Y., Ke C. (2021). Application of Metabolomics to the Discovery of Biomarkers for Ischemic Stroke in the Murine Model: A Comparison with the Clinical Results. Mol. Neurobiol..

[52] Wang Y., Wang Y., Li M., Xu P., Gu T., Ma T., Gu S. (2013). (1)H NMR-Based Metabolomics Exploring Biomarkers in Rat Cerebrospinal Fluid after Cerebral Ischemia/Reperfusion. Mol. Biosyst..

[53] Tanaka H., Yokota H., Jover T., Cappuccio I., Calderone A., Simionescu M., Bennett M.V.L., Zukin R.S. (2004). Ischemic Preconditioning: Neuronal Survival in the Face of Caspase-3 Activation. J. Neurosci..

[54] Lee T.-H., Yang J.-T., Lin J.-R., Hu C.-J., Chou W.-H., Lin C.-P., Chi N.-F. (2020). Protective Effects of Ischemic Preconditioning against Neuronal Apoptosis and Dendritic Injury in the Hippocampus Are Age-Dependent. J. Neurochem..

[55] Lin W.-Y., Chang Y.-C., Ho C.-J., Huang C.-C. (2013). Ischemic Preconditioning Reduces Neurovascular Damage after Hypoxia-Ischemia via the Cellular Inhibitor of Apoptosis 1 in Neonatal Brain. Stroke.

[56] Pinheiro D.F.d.C., Fontes B., Shimazaki J.K., Heimbecker A.M.C., Jacysyn J.d.F., Rasslan S., Montero E.F.d.S., Utiyama E.M. (2016). Ischemic Preconditioning Modifies Mortality and Inflammatory Response. Acta Cir. Bras..

[57] Liang J., Han R., Zhou B. (2021). Metabolic Reprogramming: Strategy for Ischemic Stroke Treatment by Ischemic Preconditioning. Biology.

[58] Yong M., Kaste M. (2008). Dynamic of Hyperglycemia as a Predictor of Stroke Outcome in the ECASS-II Trial. Stroke.

[59] Kwon H.-M., Lee Y.-S., Bae H.-J., Kang D.-W. (2014). Homocysteine as a Predictor of Early Neurological Deterioration in Acute Ischemic Stroke. Stroke.

[60] Lehotský J., Burda J., Danielisová V., Gottlieb M., Kaplán P., Saniová B. (2009). Ischemic Tolerance: The Mechanisms of Neuroprotective Strategy. Anat. Rec..

[61] Petras M., Tatarkova Z., Kovalska M., Mokra D., Dobrota D., Lehotsky J., Drgova A. (2014). Hyperhomocysteinemia as a Risk Factor for the Neuronal System Disorders. J. Physiol. Pharmacol. Off. J. Pol. Physiol. Soc..

[62] Williams S.R., Yang Q., Chen F., Liu X., Keene K.L., Jacques P., Chen W.-M., Weinstein G., Hsu F.-C., Beiser A. (2014). Genome-Wide Meta-Analysis of Homocysteine and Methionine Metabolism Identifies Five One Carbon Metabolism Loci and a Novel Association of ALDH1L1 with Ischemic Stroke. PLoS Genet..

[63] Kalhan S.C., Marczewski S.E. (2012). Methionine, Homocysteine, One Carbon Metabolism and Fetal Growth. Rev. Endocr. Metab. Disord..

[64] Lehotský J., Tothová B., Kovalská M., Dobrota D., Beňová A., Kalenská D., Kaplán P. (2016). Role of Homocysteine in the Ischemic Stroke and Development of Ischemic Tolerance. Front. Neurosci..

[65] Kaplan P., Tatarkova Z., Sivonova M.K., Racay P., Lehotsky J. (2020). Homocysteine and Mitochondria in Cardiovascular and Cerebrovascular Systems. Int. J. Mol. Sci..

[66] Büdy B., O’Neill R., DiBello P.M., Sengupta S., Jacobsen D.W. (2006). Homocysteine Transport by Human Aortic Endothelial Cells: Identification and Properties of Import Systems. Arch. Biochem. Biophys..

[67] Jiang X., Yang F., Brailoiu E., Jakubowski H., Dun N.J., Schafer A.I., Yang X., Durante W., Wang H. (2007). Differential Regulation of Homocysteine Transport in Vascular Endothelial and Smooth Muscle Cells. Arterioscler. Thromb. Vasc. Biol..

[68] Yu X., Huang Y., Hu Q., Ma L. (2009). Hyperhomocysteinemia Stimulates Hepatic Glucose Output and PEPCK Expression. Acta Biochim. Biophys. Sin..

[69] Tessari P., Kiwanuka E., Coracina A., Zaramella M., Vettore M., Valerio A., Garibotto G. (2005). Insulin in Methionine and Homocysteine Kinetics in Healthy Humans: Plasma vs. Intracellular Models. Am. J. Physiol.-Endocrinol. Metab..

[70] Kovalska M., Baranovicova E., Kalenska D., Tomascova A., Adamkov M., Kovalska L., Lehotsky J. (2021). Methionine Diet Evoked Hyperhomocysteinemia Causes Hippocampal Alterations, Metabolomics Plasma Changes and Behavioral Pattern in Wild Type Rats. Int. J. Mol. Sci..

[71] Kovalska M., Hnilicova P., Kalenska D., Tothova B., Adamkov M., Lehotsky J. (2019). Effect of Methionine Diet on Metabolic and Histopathological Changes of Rat Hippocampus. Int. J. Mol. Sci..

[72] Harris J.L., Choi I.-Y., Brooks W.M. (2015). Probing Astrocyte Metabolism in Vivo: Proton Magnetic Resonance Spectroscopy in the Injured and Aging Brain. Front. Aging Neurosci..

[73] Lehotsky J., Kovalska M., Baranovicova E., Hnilicova P., Kalenska D., Kaplan P., Pluta R. (2021). Ischemic Brain Injury in Hyperhomocysteinemia. Cerebral Ischemia [Internet].

[74] Kovalska M., Hnilicova P., Kalenska D., Tomascova A., Adamkov M., Lehotsky J. (2020). Effect of Methionine Diet on Time-Related Metabolic and Histopathological Changes of Rat Hippocampus in the Model of Global Brain Ischemia. Biomolecules.

[75] Role of Methionine on Epigenetic Modification of DNA Methylation and Gene Expression in Animals-ScienceDirect. https://www.sciencedirect.com/science/article/pii/S240565451730094X?via%3Dihub.

[76] Wu X., Zhang L., Miao Y., Yang J., Wang X., Wang C., Feng J., Wang L. (2019). Homocysteine Causes Vascular Endothelial Dysfunction by Disrupting Endoplasmic Reticulum Redox Homeostasis. Redox Biol..

[77] Soares D.P., Law M. (2009). Magnetic Resonance Spectroscopy of the Brain: Review of Metabolites and Clinical Applications. Clin. Radiol..

[78] Sajja B.R., Wolinsky J.S., Narayana P.A. (2009). Proton Magnetic Resonance Spectroscopy in Multiple Sclerosis. Neuroimaging Clin. N. Am..

[79] Alzheimer Disease: Postmortem Neuropathologic Correlates of Antemortem 1H MR Spectroscopy Metabolite Measurements1 | Radiology. https://pubs.rsna.org/doi/10.1148/radiol.2481071590.

[80] Unschuld P.G., Edden R.A.E., Carass A., Liu X., Shanahan M., Wang X., Oishi K., Brandt J., Bassett S.S., Redgrave G.W. (2012). Brain Metabolite Alterations and Cognitive Dysfunction in Early Huntington’s Disease. Mov. Disord..

[81] Clinical Proton MR Spectroscopy in Central Nervous System Disorders | Radiology. https://pubs.rsna.org/doi/10.1148/radiol.13130531.

[82] Faghihi R., Zeinali-Rafsanjani B., Mosleh-Shirazi M.-A., Saeedi-Moghadam M., Lotfi M., Jalli R., Iravani V. (2017). Magnetic Resonance Spectroscopy and Its Clinical Applications: A Review. J. Med. Imaging Radiat. Sci..

[83] Perła-Kaján J., Jakubowski H. (2019). Dysregulation of Epigenetic Mechanisms of Gene Expression in the Pathologies of Hyperhomocysteinemia. Int. J. Mol. Sci..

[84] Murphy E., Ardehali H., Balaban R.S., DiLisa F., Dorn G.W., Kitsis R.N., Otsu K., Ping P., Rizzuto R., Sack M.N. (2016). Mitochondrial Function, Biology, and Role in Disease. Circ. Res..

[85] Stanga S., Caretto A., Boido M., Vercelli A. (2020). Mitochondrial Dysfunctions: A Red Thread across Neurodegenerative Diseases. Int. J. Mol. Sci..

[86] Timkova V., Tatarkova Z., Lehotsky J., Racay P., Dobrota D., Kaplan P. (2016). Effects of Mild Hyperhomocysteinemia on Electron Transport Chain Complexes, Oxidative Stress, and Protein Expression in Rat Cardiac Mitochondria. Mol. Cell. Biochem..

[87] Tatarkova Z., Bencurova M., Lehotsky J., Racay P., Kmetova Sivonova M., Dobrota D., Kaplan P. (2022). Effect of Hyperhomocysteinemia on Rat Cardiac Sarcoplasmic Reticulum. Mol. Cell. Biochem..

[88] Liu M., Tang H., Nicholson J., Lindon J. (2002). Use of H-1 NMR-Determined Diffusion Coefficients to Characterize Lipoprotein Fractions in Human Blood Plasma. Magn. Reson. Chem..

[89] Kovalska M., Tothova B., Kovalska L., Tatarkova Z., Kalenska D., Tomascova A., Adamkov M., Lehotsky J. (2018). Association of Induced Hyperhomocysteinemia with Alzheimer’s Disease-Like Neurodegeneration in Rat Cortical Neurons After Global Ischemia–reperfusion Injury. Neurochem. Res..

[90] Zaragozá R. (2020). Transport of Amino Acids Across the Blood–brain Barrier. Front. Physiol..

